# Unlocking Preclinical Alzheimer’s: A Multi-Year Label-Free In Vitro Raman Spectroscopy Study Empowered by Chemometrics

**DOI:** 10.3390/ijms25094737

**Published:** 2024-04-26

**Authors:** Eneko Lopez, Jaione Etxebarria-Elezgarai, Maite García-Sebastián, Miren Altuna, Mirian Ecay-Torres, Ainara Estanga, Mikel Tainta, Carolina López, Pablo Martínez-Lage, Jose Manuel Amigo, Andreas Seifert

**Affiliations:** 1 CIC nanoGUNE BRTA, 20018 San Sebasián, Spain; e.lopez@nanogune.eu (E.L.); j.etxebarria@nanogune.eu (J.E.-E.); 2 Department of Physics, University of the Basque Country (UPV/EHU), 20018 San Sebastián, Spain; 3 Center for Research and Advanced Therapies, CITA-Alzhéimer Foundation, 20009 San Sebastián, Spain; mgarcia@cita-alzheimer.org (M.G.-S.); maltuna@cita-alzheimer.org (M.A.); mecay@cita-alzheimer.org (M.E.-T.); aestanga@cita-alzheimer.org (A.E.); mtainta@cita-alzheimer.org (M.T.); clopez@cita-alzheimer.org (C.L.); pmlage@cita-alzheimer.org (P.M.-L.); 4 IKERBASQUE, Basque Foundation for Science, 48009 Bilbao, Spain; 5 Department of Analytical Chemistry, University of the Basque Country, 48940 Leioa, Spain

**Keywords:** preclinical Alzheimer’s, cerebrospinal fluid, vibrational spectroscopy, machine learning, PLS-DA, variable selection

## Abstract

Alzheimer’s disease is a progressive neurodegenerative disorder, the early detection of which is crucial for timely intervention and enrollment in clinical trials. However, the preclinical diagnosis of Alzheimer’s encounters difficulties with gold-standard methods. The current definitive diagnosis of Alzheimer’s still relies on expensive instrumentation and post-mortem histological examinations. Here, we explore label-free Raman spectroscopy with machine learning as an alternative to preclinical Alzheimer’s diagnosis. A special feature of this study is the inclusion of patient samples from different cohorts, sampled and measured in different years. To develop reliable classification models, partial least squares discriminant analysis in combination with variable selection methods identified discriminative molecules, including nucleic acids, amino acids, proteins, and carbohydrates such as taurine/hypotaurine and guanine, when applied to Raman spectra taken from dried samples of cerebrospinal fluid. The robustness of the model is remarkable, as the discriminative molecules could be identified in different cohorts and years. A unified model notably classifies preclinical Alzheimer’s, which is particularly surprising because of Raman spectroscopy’s high sensitivity regarding different measurement conditions. The presented results demonstrate the capability of Raman spectroscopy to detect preclinical Alzheimer’s disease for the first time and offer invaluable opportunities for future clinical applications and diagnostic methods.

## 1. Introduction

Alzheimer’s disease (AD) is the primary cause of cognitive impairment and represents the main prevalent neurodegenerative disorder and challenge. Characterized by a continuum, it includes a very large preclinical (PC) stage followed by a mild cognitive impairment (MCI) period, leading ultimately to a dementia stage. The importance of early AD diagnosis cannot be overestimated. Timely and successful initiation of treatment is crucial to halting the progression of the disease [1,2,3]. Early detection primarily involves preclinical AD (PC-AD), originally defined in the late 20th century as cognitively unimpaired individuals who exhibit AD brain lesions post-mortem. With the addition of pathologic AD markers, PC-AD now includes cases in which these markers are also present in cognitively normal individuals [4,5]. To date, the European Academy of Neurology and other associations do not approve PC diagnosis in clinical practice. Currently, the diagnosis of MCI or dementia is made in clinical practice, and the recognition of PC stages is limited to the research context. Nevertheless, the relevance of early diagnosis cannot be underestimated, particularly given the potential of emerging treatments such as Lecanemab and Donanemab. These drugs promise to change the course of the disease, especially if they are used in the preclinical stages, as the ongoing AHEAD study to investigate the efficacy of Lecanemab in this context shows [6].

According to the International Working Group (IWG), “The diagnosis of AD is clinical-biological and requires the presence of both a specific clinical phenotype of AD and biomarker evidence of AD pathology” [7]. This statement makes the correct diagnosis of the disease even more difficult, as the disease is progressive and biomarkers change during years preceding the disease [8]. Its gradual progression covers a prolonged PC phase marked by sequential amyloid peptide and tau protein deposition, culminating in neurodegeneration preceding clinical symptomatology [7,9,10,11].

Although previous studies have addressed the identification of the preclinical stage, existing evidence remains limited regarding its traditional diagnostic approaches to neurological–neuropsychological assessments and the analysis of amyloid tau neurodegeneration (ATN) biomarkers (amyloid beta Aβ, phosphorylated tau and total tau) in cerebrospinal fluid (CSF) [5,12,13,14]. The ATN classification system classifies AD biomarkers into three groups, providing information on neuropathological changes [15,16,17]. AD-specific biomarkers are crucial since neurodegeneration and lesions can also occur in non-AD diseases, especially in older people with other pathologies [11,18]. Positron emission tomography (PET) imaging is used for this type of analysis; it uses radiotracers binding to Aβ or tau plaques in the brain and offers high diagnostic accuracy and localized information [19]. Another analytical route is CSF extraction by lumbar puncture and enzyme-linked immunosorbent assays (ELISA). In this case, it is possible to evaluate brain pathology and measure Aβ and tau biomarkers from the same collection. These techniques are proven, and studies have shown a strong correlation between CSF biomarkers and PET results [11,20,21]. Consequently, biomarkers are used to support the diagnosis of AD, whereas clinical diagnosis is used to identify AD severity.

**Figure 1:**
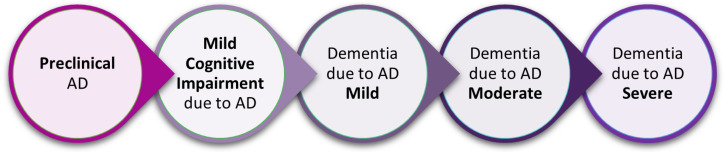
Alzheimer’s disease continuum as defined by the International Working Group (IWG).

The recent increase in imaging and fluid biomarkers of AD pathophysiology provides the opportunity to identify several biological stages in the preclinical phase of AD [22]. Positive Aβ and tau biomarkers can be observed in individuals without cognitive impairment (PC-AD), in those with MCI, and in those with dementia (Figure 1).

In the search for reliable biomarkers for neurodegenerative diseases applying novel methods, various molecular markers in tissues, biofluids, and imaging techniques are currently being investigated [23]. Although mass spectrometry (MS) and ELISA stand out as established biomarker identification and quantification techniques, they have their limitations: they are destructive, time-consuming, expensive, and require highly trained personnel [23,24].

The integration of imaging and fluid biomarkers has expanded our understanding of AD pathophysiology, offering insights into various biological stages preceding clinical symptoms. In this endeavor, novel methods are being explored, with Raman spectroscopy emerging as a holistic, more cost-effective, non-destructive, and technically less complex alternative that overcomes the limitations of conventional approaches. Raman spectroscopy is a label-free and rapid spectroscopic method that provides chemical and structural information by detecting inelastically scattered photons [25]. Moreover, it requires minimal sample preparation, eliminates the need for additional chemicals, is non-destructive, and significantly reduces analysis time. The performance of Raman spectroscopy in the classification of AD, as reported by Xu and co-authors in a comprehensive review encompassing eight selected studies [26], demonstrates high sensitivity (0.86) and specificity (0.87), indicative of its potential for future medical diagnostics.

A drawback of Raman spectroscopy with biological samples is that Raman spectra often contain non-relevant signals superimposed onto interesting ones, along with a number of artifacts (e.g., baseline drifts) that make it difficult to directly interpret the Raman peaks obtained. Therefore, it is of utmost importance to analyze Raman spectra using sophisticated machine learning methods (chemometrics) and, in particular, in combination with variable selection methods to minimize artifacts, highlight important signals and be able to build reliable classification models, which have been investigated to diagnose AD in biofluidic samples [12,27,28,29,30]. Most of the published research is based on limited statistics with small cohorts and, therefore, compromises the accuracy of classification and additional variable selection methods, providing limited insight into the physiological origin of classification results.

In this manuscript, we put forward a new and more reliable approach by combining the datasets from two cohorts from different years, thus increasing the number of participants and enhancing the robustness and reliability of the models beyond what is commonly found in the literature. The combined dataset provides significantly larger sample sizes compared to those reported in similar proof-of-concept studies utilizing both Raman spectroscopy and surface-enhanced Raman spectroscopy for the identification of AD in bodily fluids [12,27,28,29,30,31]. Although Raman spectroscopy is a very sensitive technique and susceptible to changing environmental conditions, we demonstrate here that it is possible to build stable classification models with Raman datasets from different cohorts measured during different years (2022 and 2023). The focus of this study is on the early detection of AD, i.e., the classification of preclinical Alzheimer’s, which has not yet been investigated in other studies. It is much more difficult to detect physiological changes in PC-AD, and we address this challenge with chemometric methods employing specific variable selection.

## 2. Results and Discussion

**Figure 2:**
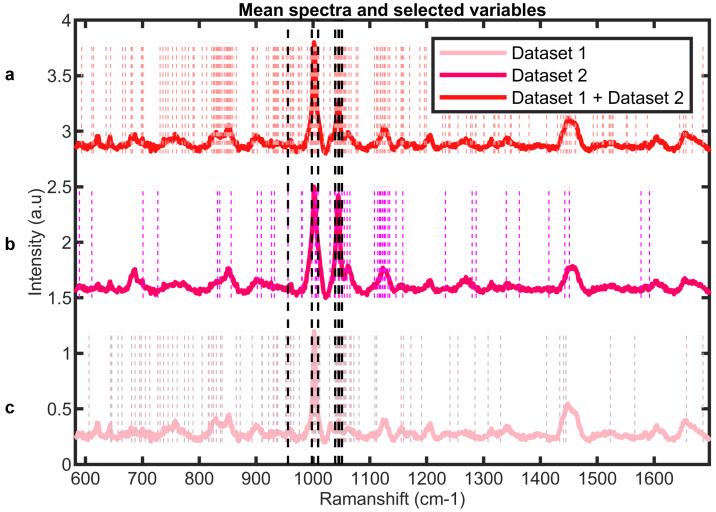
Averaged spectra and selected variables for the (**a**) Dataset 1 + Dataset 2 study. (**b**) Dataset 2 study. (**c**) Dataset 1 study. Dashed lines in black represent wavenumbers selected in common across all three datasets.

As explained in Section 3, the CSF samples were obtained from two studies performed during different years, and the Raman measurements for each of these sample sets were also performed in different years at a later time point. We refer to the corresponding datasets here as Dataset 1 and Dataset 2. Figure 2 presents the Raman spectra corresponding to Dataset 1 (light pink), Dataset 2 (dark pink), and their combined (red) form (Dataset 1 + Dataset 2), referring to the two cohorts with Raman measurements from different years. The initial focus is on variable selection for each dataset to identify molecular fingerprints indicative of PC-AD. Employing the variable selection strategy with a cross-validation of 15 random subsets and 5 iterations, Dataset 1 and Dataset 2 were reduced to 93 and 50 discriminative wavenumbers, respectively. Assembling both datasets into a unified one, the same variable selection procedure was systematically applied, discovering 213 discriminating variables. From the representative wavenumbers of each cohort, as illustrated by the dashed lines in Figure 2, distinctive and common spectral patterns emerge for the control and PC-AD groups within the Dataset 1 and Dataset 2 studies. This observation shows the potential utility of Raman spectroscopy in detecting molecular alterations associated with PC-AD. The clear differences in spectral profiles within each dataset and the presence of shared spectral features across Dataset 1, Dataset 2, and their combination indicate subtle molecular changes linked to PC-AD progression.

A more in-depth analysis of the chosen wavenumbers shows agreement with crucial peaks described in the literature as decisive for Alzheimer’s discrimination. The identified peaks, among others, are outlined in Table 1.

In particular, the specific bands at 1045 cm^−1^ and 1065 cm^−1^ exhibit significant intensity changes that correlate with alterations in amino acids attributed to AD biomarkers such as tau proteins and Aβ42 peptides [29]. These spectral peaks may correspond to taurine and hypotaurine [32], both of which are amino acid derivatives. Taurine, known for its diverse physiological functions essential for overall health and wellbeing, acts as an osmoregulatory agent [33]. Moreover, experimental studies have shown the binding of taurine with oligomeric Aβ plaques [34], preventing the neurotoxicity of Aβ and glutamate receptor agonists, which indicates a potential interaction between taurine and key pathological features associated with AD. Interestingly, the identification of characteristic bands at 727 cm^−1^ and 956 cm^−1^ shared by both guanine and phosphatidylserine (PS) highlights potential molecular mechanisms underlying AD pathology. Guanosine, known for its neuroprotective effects, is a derivative of guanine, a nucleotide base present in DNA and RNA structures. Guanine’s association with purinergic signaling and its conversion to guanosine suggests a potential link between purinergic signaling pathways and AD pathology [35]. Similarly, PS, a structural component of eukaryotic membranes, plays a multifaceted role in many biological processes, including enzyme activation, apoptosis, and neurotransmission. The dysregulation of PS and other phospholipids in AD brains alters membrane viscosity and hampers essential biological processes, potentially contributing to synaptic dysfunction and neurodegeneration [36,37].

The correlations identified in the spectral analysis highlight the complex interactions of molecular components in AD pathology and emphasize the need for comprehensive research to understand its underlying mechanisms. The subsequent deeper analysis separately evaluates machine learning models for each study (Dataset 1 and Dataset 2), as shown in Table 2. The best model, which was determined for both cohorts individually, has good performance indicators. While the Dataset 1 study demonstrates comparatively more representative features and latent variables for optimal prediction (93 features and 4 LVs for Dataset 1 and 50 features and 3 LVs for Dataset 2), it still achieves commendable accuracies of up to 0.93, although slightly below the Dataset 2 model’s accuracy of 0.97. Despite variations in the studies of individual years and the timeline of Raman measurements, the potential for PC-AD classification remains significant.

The decision to develop a unified model incorporating both studies is motivated by several factors: first, to comprehensively analyze both datasets with a larger sample size for an expected improved discrimination; second, to investigate whether combining data sets could improve the predictive abilities of the model by capturing a broader range of characteristics and patterns; lastly, to investigate common variables across the data sets and discovering common factors that clearly influence AD classification, which was triggered by the unified approach. In contrast to individual cohort-specific models, the unified model requires a higher complexity in terms of the number of latent variables for optimum performance, which is six compared to three and four LVs for individual sets Dataset 1 and Dataset 2. However, higher statistics did not improve the model’s predictive ability because of its complexity. Of course, the differences in measurement years and cohort characteristics may have influenced these outcomes. Additionally, when considering a model with variables selected jointly for all three datasets (Dataset 1, Dataset 2, and Dataset 1 + Dataset 2), a decreased discriminative power was observed, emphasizing the impact of factors such as cohort, year, or measurement strategy. The augmentation of Raman datasets by different measurement cycles, varying measurement conditions, and/or environmental changes subject to many factors will definitely increase uncertainty in a combined dataset. However, augmentation will finally lead to a stable model that considers all uncertainty factors and can be used as a clinical predictive tool. Whether the predictive power is worse or better cannot be foreseen, as long as the inner structure of such hierarchical datasets is not fully captured.

**Figure 3:**
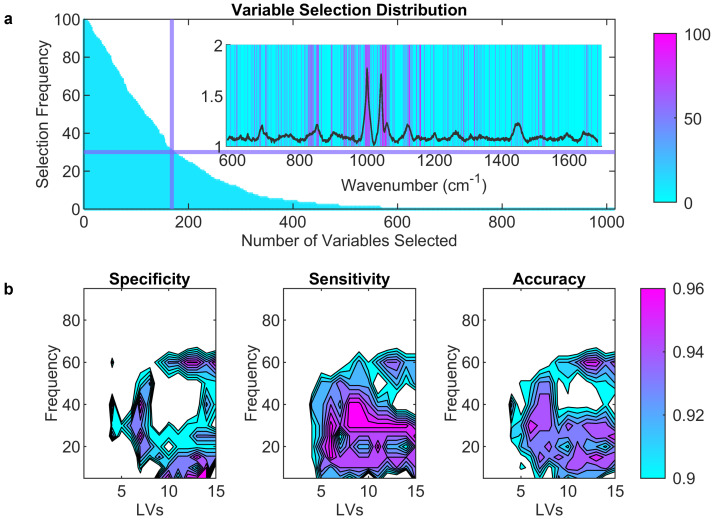
(**a**) Number of variables selected and frequency of each selected variable during the 100−fold iteration process. (**b**) Heat maps of specificity, sensitivity, and accuracy for each frequency threshold for the different models.

By extending the analysis, we employed an iterative approach for the variable selection procedure to increase discriminative information and reveal meaningful patterns in the frequency analysis of selected wavenumbers. Figure 3a shows the frequency distribution of selected wavenumbers over 100 iterations, which motivated the creation of machine learning models for various frequency thresholds. Figure 3b displays heat maps of figures of merit for all the created models and highlights the optimal PLS-DA model with six latent variables and wavenumbers for frequencies exceeding 30. This model achieves a remarkable AUC of 0.99 and an accuracy of 0.96 in predicting PC-AD cases, providing a competitive model for its statistically enhanced robustness and performance comparable to single cohorts. Iterative cross-validation or jackknifing improves the discrimination information extracted from the combined data.

**Figure 4:**
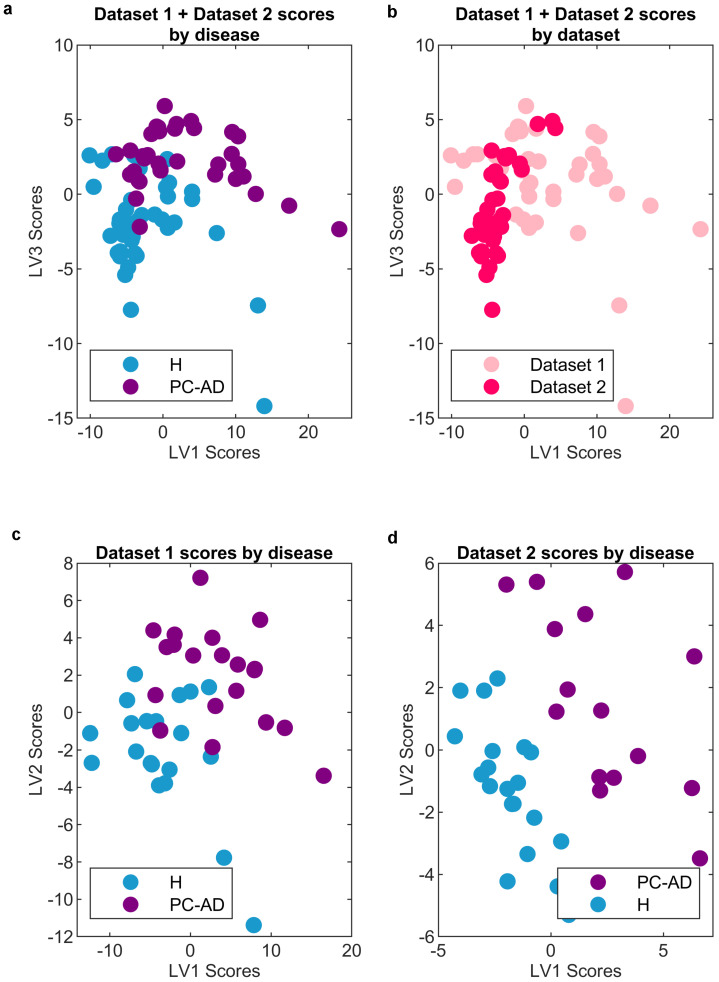
(**a**) Unified studies from Dataset 1 + Dataset 2 with disease−specific labels. (**b**) Unified studies from Dataset 1 + Dataset 2 with year−specific labels. (**c**) Dataset 1 study with disease−specific labels. (**d**) Dataset 2 study with disease−specific labels.

Alternatively, one could imagine constructing a model that only contains the variables that were selected together in all three data sets (Dataset 1, Dataset 2, and Dataset 1 + Dataset 2). However, the exclusive use of these selected wavenumbers, as shown in Table 1, proves to be insufficient for building machine learning models and leads to a decrease in classification performance. Various factors contribute to the selection of additional variables, especially cohort and measurement year. Figure 4 shows the model scores derived from Table 2, thereby providing a visual representation. The two-latent space depicted in the upper part of Figure 4, which includes LV1 and LV2, highlights the models’ discriminative capacity for PC-AD in both studies on Dataset 1 and Dataset 2. In particular, the color variation in the labels, as observed in the lower part of Figure 4, indicates clustering between healthy and preclinical groups, whereas the studies from Dataset 1 and Dataset 2 exhibit clustering in a different direction. This explains why more variables and model latent spaces are required when combining the studies from Dataset 1 and Dataset 2 to build a more robust and predictive model for the discrimination of PC-AD.

## 3. Materials and Methods

### 3.1. Dataset Creation

The samples were collected from a population-based clinical–biological cohort of adults with and without cognitive decline. They belonged to a cross-sectional study, and the recruitment of all individuals was performed by the CITA-Alzheimer Foundation. The syndromic diagnosis was established through comprehensive neurological and neuropsychological assessment, structural magnetic resonance neuroimaging (MRI), and a CSF AT(N) biomarkers study. CSF samples were obtained in 2014 and 2015 (Dataset 1) from the participants of the Gipuzkoa Alzheimer Project (GAP) [38] and from 2016 to 2018 (Dataset 2) in the DEBA study [39]. Research was conducted in accordance with the Declaration of Helsinki and approved by local Ethics Committees. A total of 75 volunteers were recruited to detect PC-AD using CSF samples. In both studies, volunteers were categorized into a control group comprising healthy individuals (H) and PC-AD patients who exhibited abnormal biomarker values while maintaining normal cognitive function. The control group was defined by specific CSF analysis cutoff values (Aβ42 > 1030 pg/mL, total-Tau > 300 pg/mL, p-Tau > 27 pg/mL) to validate ATN negativity and cognitive normalcy. The inclusion of data from two separate studies increases robustness and provides an extended time range for the analytical framework, enabling a comprehensive assessment of preclinical AD detection performance. Age and gender distribution were comparable between the control and preclinical AD groups, as summarized in Table 3.

### 3.2. Raman Measurements and Sample Preparation

**Figure 5:**
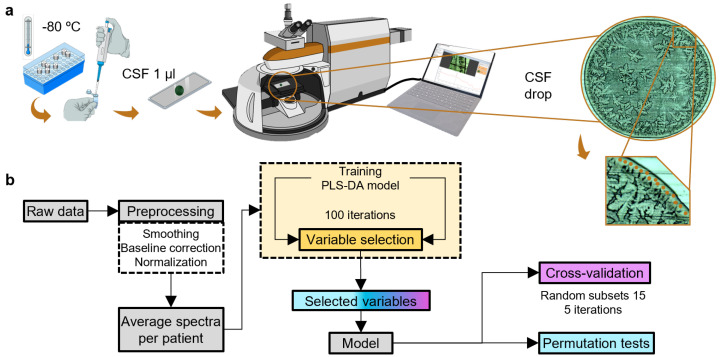
(**a**) Sample preparation and Raman measurement conditions. (**b**) Stages of the machine learning workflow.

CSF samples were collected according to international consensus recommendations [21] and centrifuged immediately at 4 °C and stored at −80 °C within one hour of lumbar puncture. For Raman measurements, CSF samples underwent ultracentrifugation using an Amicon^®^ Ultra-0.5 filter with a 3 kDa pore size, resulting in a protein-rich supernatant. Raman measurements were performed with an inVia Qontor confocal Raman microscope (Renishaw plc, Wotton-under-Edge, UK). One microliter of the CSF sample was deposited onto a microscope glass slide covered with aluminum foil to enhance the Raman signal. The droplet was dried under vacuum for 10 min before each Raman measurement. Optimal measurement conditions were defined for a balanced signal-to-noise ratio and sample preservation. Point-by-point mapping was employed, capturing 15 spectra at the ring of the dried droplet (Figure 5). Laser wavelength and output power were set to 785 nm and 73 mW, respectively; a 50×-long distance objective was selected, and 50 accumulations were performed with an exposure time of 1 s. Raman measurements from Dataset 1 samples were taken in 2022 and for the Dataset 2 samples in 2023.

### 3.3. Modeling Workflow

#### 3.3.1. Data Preprocessing

**Figure 6:**
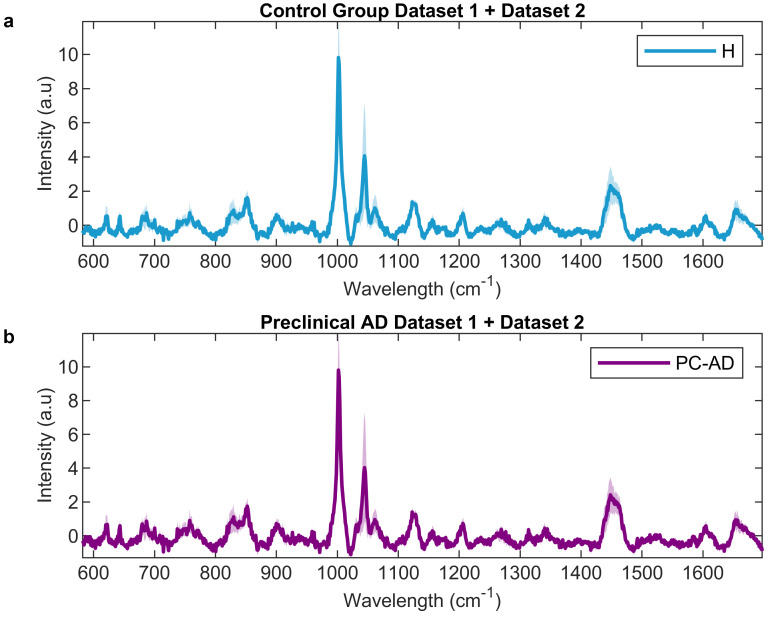
Preprocessed and averaged spectra per patient to unify both cohorts from Dataset 1 and Dataset 2; shaded area refers to the standard error. (**a**) Healthy or control group. (**b**) Preclinical Alzheimer’s subjects.

All stages of the data analysis were carried out in MATLAB 2022a (The MathWorks, Inc., Natick, MA, USA) using in-house routines and the PLS-Toolbox (Eigenvector Research Inc., Wenatchee, WA, USA). The detailed workflow, covering feature extraction, model training, and validation, is explained in the following section. The order of these steps (Figure 5b) is essential and was carefully considered in the spectroscopic data analysis workflow. Figure 5b provides a visual summary of the intrinsic process steps of the data analysis approach. The Raman spectra underwent a meticulous pipeline for optimum preprocessing, which is important, non-trivial, and can significantly affect model performance [40]. The data collected from the two cohorts, Dataset 1 and Dataset 2, exhibit similar and distinct characteristics. To ensure meaningful comparisons and to facilitate the creation of a unified model incorporating both cohorts, a consistent preprocessing strategy was applied, starting with noise reduction and signal enhancement (using Savitzky-Golay filtering), followed by baseline correction using Whittaker’s method to eliminate fluctuations and artifacts and standard normal variate (SNV) spectral scaling [41,42,43]. All 15 spectra per patient were first preprocessed and scaled and subsequently averaged per patient [44], as depicted in Figure 6, to obtain one representative spectrum per patient for subsequent variable selection and classification. Figure 6 displays the mean spectra for healthy (upper) and PC-AD (lower) individuals, accompanied by the standard error (shadowed area). The subtle differences between healthy and preclinical subjects are barely visible, and classification can only be achieved by proper selection of feature extraction and subsequent machine learning algorithms.

#### 3.3.2. Feature Extraction Methods for Identification of Discriminative Molecules in Preclinical AD’s Discrimination

Feature extraction was performed using the integrated action of variable importance in projection (VIP) method and the selectivity ratio (SR). This approach uses the spectra projected into the latent space of a partial least squares discriminant analysis (PLS-DA) model to select iterative variables due to their importance in the model [45]. The iterative process optimizes wavenumber subsets by comparing the root mean square error of cross-validation (RMSECV) values and removing those wavenumbers with the lowest influence on prediction. The approach integrates VIP and SR, progressively eliminating variables until the model no longer improves. This variable selection strategy not only elevates the importance of the feature set but also plays a crucial role in identifying relevant wavenumbers to distinguish between distinct patient cohorts. Selected wavenumbers serve as valuable indicators, which help identify molecular vibrations that contribute to the differentiation of PC-AD. To assess the reliability of the selected features, an iterative approach was implemented by running the variable selection strategy 100 times. In each iteration, 10 patients (5 from each class) from a dataset of 75 patients were randomly excluded, allowing for both interclass and intraclass variability. The procedure was then applied with the same cross-validation structure of 15 random subsets and 5 iterations, ensuring a robust assessment by excluding 10 different patients from model training and cross-validation in each iteration. Subsequently, the variable selection distribution across the 100 iterations was examined, and an additional model based on this analysis was constructed, as shown in the results section.

#### 3.3.3. PLS-DA Model Development and Evaluation Metrics

Following the workflow from Figure 5 and using the reduced feature set from the variable selection procedure, reliable PLS-DA models were constructed. PLS-DA is a versatile multivariate classification method selected for its simplicity and effectiveness [46]. Differentiating from classical PLS regression, PLS-DA involves an additional step where a suitable threshold is applied to the computed y values. This thresholding process aids in determining the classification of a sample within a specific class. It is a powerful tool for modeling the relationship between variables and reducing data dimensionality while preserving the covariance structure, making it particularly relevant to our study. The optimization process focuses on tuning the number of latent variables (LVs). PLS-DA is well-suited for handling complex data relationships and correlated variables, making it an ideal choice for efficient machine learning [46,47,48,49]. Due to the low number of subjects in each of the individual cohorts and to ensure the robustness of the model, a cross-validation approach with random subsets was adopted, in which each data set was divided into 15 distinct subsets with 5 iterations. This resampling strategy of cross-validation included partitioning the dataset into training sets and diverse cross-validation sets to evaluate the model performance [44,50]. The performance of the PLS-DA models was systematically evaluated using standard classification metrics, including accuracy, sensitivity, specificity, and the area under the receiver operating characteristic curve (AUC-ROC). Permutation tests were also performed to determine the stability of the models. These tests involved randomizing the assignment of class labels and re-evaluating the models multiple times to assess the likelihood of obtaining similar performance by chance [44,51,52]. The permutation tests provided valuable statistical information on the reliability and stability of the developed PLS-DA models.

## 4. Conclusions

Our study explores the potential of Raman spectroscopy in combination with advanced chemometric methods as an innovative and far less complex technical approach for the early diagnosis of preclinical Alzheimer’s disease compared to positron emission tomography, computed tomography, or CSF analysis. The priority of early detection results from the prolonged preclinical phase of Alzheimer’s disease, in which medication and the development of new therapies could help slow down the pathogenesis. Traditional diagnostic modalities, such as neurological–neuropsychological assessments and biomarkers of cerebrospinal fluid, have limitations and require the exploration of alternative methods. Raman spectroscopy provides a molecular fingerprint of physiology without subjective interpretations and relatively simple technical effort. In our study, we investigated dried droplets of cerebrospinal fluid and took Raman measurements at the ring of the dried structures. Our investigation, across multiple years of sampling, includes two separate studies and corroborates the potential of Raman spectroscopy to distinguish between healthy subjects and those in the preclinical stage of Alzheimer’s with high accuracies reaching 0.96 in a cross-validated model. We demonstrated significant discriminative power despite variations in cohorts and measurement years. Fusing the data from both studies not only improves the robustness of the overall model but also allows for a more comprehensive assessment of the variables that play a role in the classification of preclinical Alzheimer’s disease. Significant identified wavenumbers were consistent with key peaks for Alzheimer’s disease reported in the literature, including amino acids found in established biomarkers such as tau proteins and Aβ42 peptides. Our study represents a significant advance in the application of Raman spectroscopy for the early detection of Alzheimer’s, and we note that the influence of cohort-specific factors, including sampling and measurements at different time stamps and under different conditions, underlines the need for further research and larger datasets to capture as much uncertainty as possible, incorporate the full range of various inter and intraclass variabilities, and ultimately, provide a general robust and reliable prediction model for new, unseen data in clinical settings via external validation.

## Figures and Tables

**Table 1:** Characteristic molecular vibrations for discriminating PC-AD extracted from Raman spectra.

Wavenumber (cm^−1^)	Biomarkers	Description [32]
727	Nucleic acids	Phosphatidylserine, hypotaurine, guanine
956	Proteins, carbohydrates	$\nu_{1}$ of the phosphate group, guanine
998		Monophosphate group
1009	Phenylalanine	Tryptophan
1039	Proteins, carbohydrates	Taurine
1045		Hypotaurine
1046		Hypotaurine, taurine
1051		Taurine
1065		Hypotaurine

**Table 2:** Figures of merit for PLS-DA models for preclinical AD prediction across different datasets. Dataset 1 and Dataset 2 individually; variable selection across the whole dataset: Dataset 1 + Dataset 2; shared variables in all datasets: Dataset 1 + Dataset 2 in common; variables occurring more than 30 times across 100 iterations for the combined dataset: Dataset 1 + Dataset 2 thr@30.

Cohort	Matrix	AUC	Accuracy	Sensitivity	Specificity	LVs	Variables
Dataset 1	40 × 93	0.99	0.93	0.95	0.92	4	93
Dataset 2	35 × 50	1.00	0.97	0.93	0.98	3	50
Dataset 1 +	75 × 213	0.98	0.93	0.91	0.94	6	213
Dataset 2 Dataset 1 + Dataset 2 in common	75 × 9	0.61	0.53	0.63	0.51	2	9
Dataset 1 + Dataset 2 thr@30 ^1^	75 × 168	0.99	0.96	0.93	0.96	6	168

^1^ Variables occurring more than 30 times over the process of 100 iterations.

**Table 3:** Datasets utilized in the study: Dataset 1 corresponds to samples collected between 2014 and 2015, while Dataset 2 corresponds to a cohort with samples collected between 2016 and 2018.

	Status	Total		Male/Female	Age
Dataset 1	Healthy	20	40	10/10	59.5 ± 6.8
	Preclinical	20		10/10	65.4 ± 5.1
Dataset 2	Healthy	20	35	11/9	65.7 ± 6.1
	Preclinical	15		10/5	68.5 ± 6.2
Dataset 1 +	Healthy	40	75	21/19	62.7 ± 7.1
Dataset 2	Preclinical	35		20/15	66.7 ± 7.7

## Data Availability

The data presented in this study are available on request from the corresponding author.

## Abbreviations

The following abbreviations are used in this manuscript:

AD	Alzheimer’s disease
PC	Preclinical
MCI	Mild cognitive impairment
PC-AD	Preclinical Alzheimer’s disease
IWG	International working group
ATN	Amyloid, tau, and neurodegeneration
Aβ	Amyloid beta
CSF	Cerebrospinal fluid
PET	Positron emission tomography
ELISA	Enzyme-linked immunosorbent assays
MS	Mass spectrometry
MRI	Magnetic resonance imaging
GAP	Gipuzkoa Alzheimer project
H	Healthy individuals
SNV	Standard normal variate
VIP	Variables in projection
SR	Selectivity ratio
PLS-DA	Partial least squares discriminant analysis
RMSECV	Root mean square error of cross-validation
LV	Latent variable
AUC-ROC	Area under the receiver operating characteristic curve
PS	Phosphatidylserine
